# Microvascular decompression in trigeminal neuralgia - a prospective study of 115 patients

**DOI:** 10.1186/s10194-022-01520-x

**Published:** 2022-11-19

**Authors:** Anne Sofie Schott Andersen, Tone Bruvik Heinskou, Per Rochat, Jacob Bertram Springborg, Navid Noory, Emil Andonov Smilkov, Lars Bendtsen, Stine Maarbjerg

**Affiliations:** 1 Danish Headache Center, Department of Neurology, Copenhagen University Hospital, Rigshospitalet – Glostrup, Valdemar Hansens Vej 5, 2600 Glostrup, Denmark; 2 Department of Diagnostic Radiology, Copenhagen University Hospital, Rigshospitalet – Glostrup, 2600 Glostrup, Denmark; 3 Department of Neurosurgery, Copenhagen University Hospital, Rigshospitalet – Blegdamsvej, 2100 Copenhagen, Denmark

**Keywords:** Neurosurgery, Outcome, Complication

## Abstract

**Background:**

Trigeminal neuralgia is a severe facial pain disorder. Microvascular decompression is first choice surgical treatment of patients with classical TN. There exist few prospective studies with an independent evaluation of efficacy and complications after MVD.

**Objectives:**

We aimed to assess outcome and complications after microvascular decompression from our center.

**Methods:**

We prospectively recorded clinical characteristics, outcome, and complications from consecutive patients with either classical or idiopathic (only patients with a neurovascular contact) trigeminal neuralgia undergoing microvascular decompression. Neurovascular contact was evaluated by 3.0 Tesla MRI. Patients were assessed before and 3, 6, 12, and 24 months after surgery by independent assessors.

**Results:**

Of 115 included patients, 86% had a clinically significant outcome (i.e., BNI I – BNI IIIb). There was a significant association between an excellent surgical outcome and the male sex (OR 4.9 (CI 1.9–12.8), *p* = 0.001) and neurovascular contact with morphological changes (OR 2.5 (CI 1.1–6.0), *p* = 0.036). Significantly more women (12/62 = 19%) than men (2/53 = 4%) had a failed outcome, *p* = 0.019. The most frequent major complications were permanent hearing impairment (10%), permanent severe hypoesthesia (7%), permanent ataxia (7%), and stroke (6%). Most patients (94%) recommend surgery to others.

**Conclusion:**

Microvascular decompression is an effective treatment for classical and idiopathic (only patients with a neurovascular contact) trigeminal neuralgia with a high chance of a long-lasting effect. The chance of an excellent outcome was highest in men and in patients with classical trigeminal neuralgia. Complications are relatively frequent warranting thorough patient evaluation and information preoperatively.

**Trial registration:**

Clinical.trials.gov registration no. NCT04445766.

**Supplementary Information:**

The online version contains supplementary material available at 10.1186/s10194-022-01520-x.

## Introduction

Trigeminal neuralgia (TN) is characterized by severe, shock-like trigger-evoked pain paroxysms in the distribution of the trigeminal branches. The pain is debilitating physically, mentally and socially. Women are more commonly affected than men. Incidence increases with age, and the average age of TN onset is 53 years [1, 2].

TN is classified in classical, idiopathic, and secondary form, the latter when it is caused by an underlying disease such as multiple sclerosis or a space-occupying lesion. Studies have shown a strong association between neurovascular contact (NVC) with morphological changes of the trigeminal nerve and TN [3–6].. In classical TN, there is NVC with morphological changes of the nerve on the symptomatic side, whereas in idiopathic TN, there is either a NVC without morphological changes of the trigeminal nerve or no NVC [7, 8]. Approximately 50% of patients with TN have a NVC with morphological changes, i.e. classical TN [4]. The study by Hughes et al. supports these findings [6].

First-line medical treatments for TN are the sodium channel blockers carbamazepine and oxcarbazepine, the latter showing higher tolerability [9]. Gabapentin, pregabalin, or lamotrigine can be used either as add-on or as monotherapy should the first line medication not be sufficient However, their utility is often limited by side effects such as dizziness, tiredness and double vision, [9] which is seen in 50% of the patients [10].

Neurosurgery is performed in approximately 30% of patients with TN followed at the Danish headache Center because of insufficient medical effect or unacceptable medical side effects [11]. In line with the European management guidelines, we consider microvascular decompression (MVD) first-choice surgical treatment in medically refractory classical TN and a potential first-choice treatment in medically refractory patients with idiopathic TN with idiopathic TN *with* NVC [9]. For patients with idiopathic trigeminal neuralgia *without* any NVC, we only consider neuroablative procedures. We never consider MVD in MRI negative patients.

The purpose of MVD is to alleviate the vascular compression of the trigeminal nerve. According to previous studies of MVD, 71–80% of patients are completely pain free at long-term follow-up with better outcome for patients without concomitant continuous pain [12, 13]. MVD implies delicate posterior fossa surgery with partial exposure of the cerebellum, brainstem and cranial nerves, and therefore carries a risk of complications such as cranial nerve dysfunction, aggravation of pain, meningitis, leakage of cerebrospinal fluid, stroke, and ultimately death [14].

Previous studies on outcome and complications after MVD are hampered by methodological shortcomings such as retrospective methodology, inadequate use of diagnostic criteria, and non-independent evaluation of outcome and complications [15, 16]. Few other prospective studies on effect and complications after MVD exists [17, 18]. This study is among the first studies to prospectively report effect and complications 24 months after MVD using independent assessors.

## Methods

### Definition of the cohort

This prospective, observational single-center study evaluated patients with classical or idiopathic (only patients with a neurovascular contact) TN from the Danish Headache Center (DHC), a tertiary medical treatment centre for headache and facial pain. The diagnosis of TN was based on the International Classification of Headache Disorders editions ICHD-2, ICHD-3-beta and ICHD-3 respective to the time of inclusion [8, 19, 20].

The study cohort included patients referred from DHC to the Department of Neurosurgery, Rigshospitalet, Copenhagen and treated with MVD between May 2012 and October 2018. The number of patients included in the inclusion period determined the sample size. The methodology of the data collection was largely the same as in our previously published studies [4, 21] Inclusion criteria were: a diagnosis of classical or idiopathic (only patients with a neurovascular contact) TN verified by a neurologist preoperatively, a 3.0 Tesla MRI according to a pre-defined protocol [4], a pre-operative evaluation by a neurologist at DHC, at least 24 months follow-up of effect and complications by neurologist at the DHC, and informed patient consent. Exclusion criteria were a) previous MVD, and b) psychiatric or mental illness that hindered informed consent.

### Neurological assessments before microvascular decompression

All patients were seen by a neurologist at DHC before referral for neurosurgery. The neurologist evaluated the diagnosis and conducted a standardized purpose-built semi-structured interview [15]. Patients were asked about previous and current treatments with respect to medication, dosages and side effects, and previous neurosurgical intervention(s). Clinical and neurological examination was part of the initial assessment. The patients were treated medically according to the guidelines from the European Academy of Neurology [9], with the exemption that we tried out the most efficacious of carbamazepine and oxcarbazepine with an add on of gabapentin, pregabalin or lamotrigine before considering referral to neurosurgery [11, 22]. We have previously demonstrated that the add-on treatment strategy before referral to neurosurgery, did not lead to a significant delay of surgery [11].

### Neuroimaging before microvascular decompression

All patients had MRI before neurosurgical referral. We used a 3.0 T Phillips Achieva imager (Phillips Medical Systems). The MRI-protocol was described in detail previously [4]. It involved the following sequences: T2-weighted DRIVE SPIR, T2-weighted turbo-spin-echo and 3D balanced fast field echo (BFFE) and 3D time of flight magnetic resonance angiography (s3DI MC HR).

The MRI was evaluated blinded to the pain side. The MRI was evaluated for the presence of NVC. The evaluation was done by a two neuroradiologist and based on visual inspection with measurement. NVC was evaluated for presence of morphological changes of the trigeminal nerve. NVC was defined as a contact between any blood vessel and the trigeminal nerve without visible cerebrospinal fluid between the two structures [4]. Morphological changes was defined as a contact causing compression, displacement, distortion, indentation and/or atrophy of the trigeminal nerve [4, 8]. Postoperative MRI was only performed when there was a relevant clinical indication.

All patients were offered the option of a neurosurgical consultation to discuss neurosurgical treatment options [11]. Only patients with NVC were offered MVD, i.e. both patients with classical TN (NVC with morphological changes) and the subgroup of idiopathic TN patients with NVC without morphological changes). Patients without NVC were offered the ablative procedures balloon compression or glycerol injection [21]. The neurosurgeon was the key decider on which neurosurgical procedure to offer the patient.

### Neurosurgical technique

**Fig. 1:**
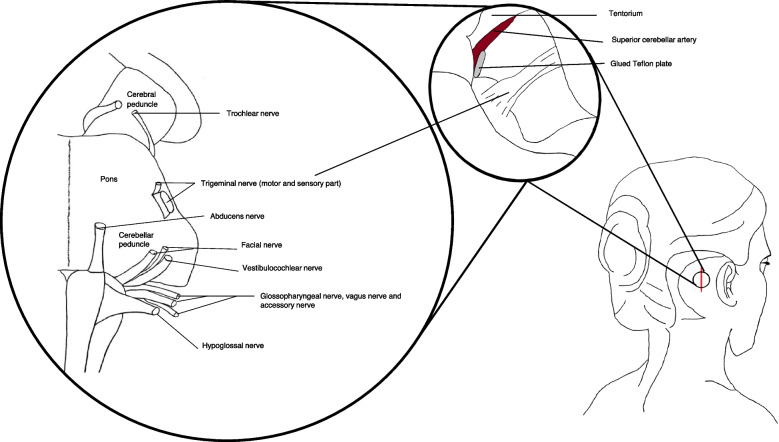
Illustration of the principles of microvascular decompression and the cranial nerves in proximity to the surgical area. The anatomical localization of the entry of the cranial nerves into the brainstem and the surgical field of microvascular decompression. The procedure was performed with the patient in a park bench position under general anaesthesia via an approximately 2 × 3 cm retrosigmoid craniectomy. Via a supracerebellar infratentorial approach, the cerebellopontine angle was visualized, and the trigeminal nerve and compressing vessel(s) were identified (Fig. 1). If the superior cerebellar artery was causing the compression, the nerve was alleviated by transposition of the blood vessel towards the tentorium, where it was fixed with Teflon and glue. If the compression was caused by the posterior or anterior inferior cerebellar artery the blood vessel was transposed caudally and fixed with Teflon if possible. If the surgeon was unable to transpose the artery, a piece of Teflon was interposed between the trigeminal nerve and the conflicting artery. If a vein was causing the compression, the vein was either divided to avoid avulsion or if possible, a piece of Teflon was interposed between the trigeminal nerve and the vein. The surgeon preferred not to coagulate veins and in particular sought to preserve the superior petrosal vein. Surgery was performed without the use of neuronavigation or brainstem auditory evoked responses or other neuromonitoring. Cerebellar retraction was not used, and neither were specific relaxation techniques. All procedures were performed microscopically

The MVD was conducted as a modified Jannetta procedure [23] and is shown and described in Fig. 1. The general technique is described in detail elsewhere [23]. The procedure was performed by one of three surgeons (JB, PR and JS) from the Department of Neurosurgery, Rigshospitalet. The surgeon registered if any perioperative complications had happened. Patients treated with platelet inhibitors or anticoagulant were required to pause the medication before the procedure, and on the day of surgery the applicable analysis (Multiplate® platelet function analysis or thromboelastography (TEG) as was performed when considered relevant. After surgery, patients were typically admitted for 2–3 days at the neurosurgical department. Here, the patients received guidance on how to taper off current TN medication.

### Follow-up after microvascular decompression

Patients were prospectively assessed by a neurologist at 3, 6, 12 and 24 months after MVD, either at out-patient visits or by telephone interviews. Outcome and complications were evaluated based on standardized follow-up schemes, questionnaires, and physical examinations. All assessments were conducted by independent assessors, i.e., neurologists from the DHC. The assessors were clinically working neurologists at the DHC and not (except Lars Bendtsen) authors of this paper. The standardized follow-up scheme included details on current pain intensity, effect of MVD according to a modified Barrow Neurological Institute (BNI) pain scale, surgical complications and current use of medication and medical side effects.

A self-complete questionnaire was mailed (with a return envelope) to the patients 24 months after MVD. A reminder was mailed after a couple of months if we had not received any answer. The questionnaire (Additional file 1: Supplementary material A) included 31 quantitative and qualitative questions concerning effect of MVD according to BNI, pain intensity, current medications, surgical complications related, and patient satisfaction after MVD. If there were disagreement between the self-complete questionnaire and the standardized follow-up scheme, data from the standardized follow up scheme was reported in this study. Patient satisfaction levels were registered on a 7-point Likert scale, anchored at 1 = very dissatisfied and 7 = very satisfied. Scores of 1–3 was labelled as dissatisfied, a score of 4 as neither satisfied nor dissatisfied and scores 5–7 as satisfied. The patients were also asked if they would recommend MVD to others. The questionnaire was developed by Zakrzewska et al. [24] and modified and translated by the TN research group at DHC [22].

The BNI assessments were completed in cooperation with the patient at the follow-up visits and by the patient alone in the self-complete questionnaire.

### Outcome

The primary outcomes were pain relief and complication rate after 24 months.

### Definition of primary outcome

Pain relief was registered on a modified BNI pain scale with the same categories used in a previous study [22]. The BNI categories were grouped according to four overall neurosurgical outcomes. The categories are shown in Table 2. We defined a ‘clinically significant outcome’ as BNI I - BNI IIIB.

### Definitions of complications

Surgical complications were predefined in the study protocol. Complications were subgrouped as major and minor complications and catogorized into transient or permanent (Table 3 and Table 4). Hearing and vision impairment were verified by an otologist and ophtalmologist, respectively. In case of a stroke, the resulting possible cranial nerve dysfunction, dizziness or ataxia, was *not* included as separate major or minor complications. Patients with strokes were assesed by the modified Rankin Scale to measure the degree of disability and dependence after a stroke. Each major complication was described in detail (Additional file 2: Supplementary material B).

### Definitions of recurrence of pain

Recurrence of pain was defined as recurrence of pain at 24 months assessment for a patient who was completely pain-free (BNI I) 12 months after MVD. Minor recurrence was defined as partial pain relief at 24 months follow-up where the pain was adequately controlled with medical treatment (BNI II-IIIB). Major recurrence was defined as recurrence of pain at 24 months follow-up, that was not adequately controlled with medical treatment (BNI IV-VB).

### Statistical analyses

Continuous and ranked data are summarized by descriptive statistics. Categorical variables are presented with frequency distributions (N, %), reported as numbers, means, percentages and with 95% confidence intervals (CI). A chi square test was used to test associations between independent categorical variables. Multiple logistic regression analysis with backward stepwise elimination was used to test for associations between a subset of predefined clinical characteristics and excellent outcome of MVD. The predefined variables were; sex (men vs. women), NVC with morphological changes found on MRI (yes vs. no), TN with purely paroxysmal pain vs. with concomitant continuous pain, age at time of MVD (below vs. over 70 years), and disease duration (below vs. above 2 years) at time of MVD. The variables were retained in the model if the association was significant (*p* < 0.05) and variables with no significant association were excluded sequentially.

As previously reported by our group, the odds for an excellent outcome after MVD are higher in men compared to women and NVC with morphological changes are significantly more prevalent in men [21, 25]. The regression model therefore included the interaction between sex and neurovascular contact with morphological changes.

If more than 10% of the clinical data were missing, the patient was excluded from the analyses. Missing data was considered missing at random. *P*-values was reported as two-tailed with a level of significance of 5%. Analyses were carried out using SAS 9.4 (SAS Institute Inc., NC, USA). We used the STROCSS reporting guidelines.

## Results

**Fig. 2:**
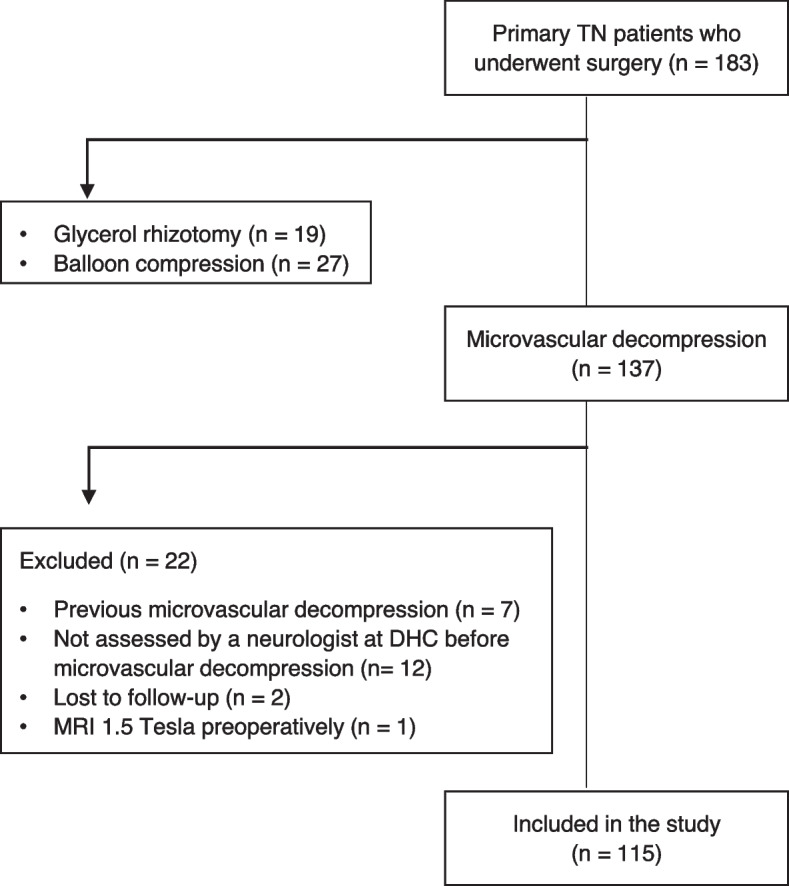
Flowchart of inclusion of patients with trigeminal neuralgia. *TN* trigeminal neuralgia, *DHC* Danish Headache Center

In total, 183 patients had surgical treatment for TN in the inclusion period, out of which 137 patients had MVD (Fig. 2). Twenty-two patients were excluded and therefore 115 patients were included in this study. Clinical characteristics and demographics are presented in Table 1. As there was too much missing data from the 3- and 6-months assessments it is not reported.

**Table 1:** Demographics and clinical characteristics of included patients with trigeminal neuralgia (*n* = 115)

	N (%)	95% CI
**Demographics**		
Male	53 (46)	37–56
Female	62 (54)	44–63
Age at TN onset, mean, years	55.4	53.0–57.8
Age at operation, years	62.0	59.6–64.4
Above 70 years of age at operation	44 (38)	29–47
Duration of TN at time of operation, mean and range, years	7.1	0–40
Long duration (> 2 years) of TN at time of surgery	96 (83)	75–90
**Clinical characteristics**		
Right-sided pain	67 (58)	49–67
Left-sided pain	48 (42)	33–51
Bilateral pain	0	–
Concomitant continuous pain^a^	61 (54)	44–63
Classical TN	70 (61)	51–70
Idiopathic TN	45 (39)	30–49
Periods of remission	60 (55)	45–64
V1	5 (4)	1–10
V2	16 (14)	10–24
V3	26 (23)	16–32
V1 + V2	13 (11)	6–19
V2 + V3	39 (34)	26–44
V1 + V2 + V3	16 (14)	8–22
Sensory abnormalities at neurological examination^a^	45 (40)	31–49
History of ablative neurosurgical procedures	1 (1)	0–5
Response to sodium channel blockers	101 (88)	80–93

Values represent numbers of patients (%) unless otherwise indicated. *CI* confidence interval, *TN* trigeminal neuralgia

^a^Only patients without a history of previous trigeminal neuralgia surgery were included in the analyses (*n* = 114)

### Outcome 24 months after microvascular decompression

Ninety-nine (86%) patients had a clinically significant outcome, defined as an excellent or good outcome (BNI I-IIIB), 24 months after MVD (Table 2). Eighty (70%) patients had an excellent outcome (BNI I). In two (2%) patients the outcome was poor (BNI IV). In 14 (12%) patients, the outcome was considered as failure, either due to no pain relief (BNI VA) (*n* = 5), aggravation of pain (BNI VB) (*n* = 2), or subsequent neurosurgical treatment before the 24 months follow-up (*n* = 7).

**Table 2:** Outcome at 24 months according to the seven-scale modified Barrow Neurological Insitute pain intensity score (BNI) and the corresponding neurosurgical outcome (*n* = 115)

Score	Pain description	n (%)	Neurosurgical outcome
BNI I	Complete pain relief: No pain and no medication	80 (70)	Excellent
BNI II	Partial pain relief: Occasional pain but no medication required	12 (10)	Good
BNI III A	Partial pain relief: No pain but daily medication required	3 (2)	Good
BNI III B	Partial pain relief: Occasional pain but adequately controlled with medication	4 (3)	Good
BNI IV	Poor pain relief: reduced pain but not adequately controlled with medication	2 (2)	Poor
BNI V A	No pain relief	5 (4)	Failure
BNI V B	Aggravation of pain	2 (2)	Failure
	Subsequent neurosurgical treatment before 24 months follow-up	7 (6)	Failure
Total		115	

A clinically significant outcome = BNI I-BNI IIIB = Surgical outcome of both excellent and good

### Recurrence

Eighty-seven (76%) patients had an excellent surgical outcome at the 12-months follow-up, and of those nine (10%) patients had minor recurrence (BNI II-IIIB) and four (5%) patients had major recurrence (BNI IV-VB) at the 24-monts follow-up. In six patients the outcome went from being BNI II-IIIB to BNI I between 12- and 24-months follow-up.

### Prognostic factors

Twelve (86%) of the 14 patients with a surgical outcome of failure were women. Significantly more women (12/62 = 19%) than men (2/53 = 4%) had a failed outcome (*p* = 0.019). Conversely, significantly more men had an excellent outcome (46/53 = 87%) compared to women (34/62 = 55%), *p* ≤ 0.005. Significantly more men (51/53 = 96%) than women (48/62 = 77%) had a clinically significant outcome (*p* = 0.006).

The chance of an excellent outcome was significantly higher in classical TN (56/70 (80%)) compared to idiopathic TN (24/45 (53%)), *p* = 0.005. and the associated OR was 2.5 (CI 1.1–6.0), *p* = 0.036. There was no significant difference in the number of patients with a clinically significant outcome among patients with classical TN (62/70 (89%)) and idiopathic TN (37/45 (82%), *p* = 0.494. When looking at women only, there was no statistically significant difference in the number of women with classical TN and an excellent outcome (22/33 = 67%) compared with women with idiopathic TN and an excellent outcome (12/29 = 41%), *p* = 0.073. When looking at men separately, there was no statistically significant difference in the number of men with classical TN and an excellent outcome (33/37 = 89%) compared with men with idiopathic TN and an excellent outcome (13/16 = 81%), *p* = 0.419.

For the logistic regression model, only two variables were retained in the reduced model as classical TN (OR = 2.5 (CI 1.1–6.0), *p* = 0.036) and the male sex (OR 4.9 (CI 1.9–12.8), *p* = 0.001) significantly predicted a clinically relevant outcome. The other variables included in the full model (concomitant continuous pain vs. purely paroxysmal pain, above vs. below 70 years at time of surgery, disease duration above vs. below 2 years and the interaction between sex and neurovascular contact with morphological changes were not significant.

### Complications

The major and minor complications are presented in Table 3 and Table 4. At 12 months follow-up, 33 (29%) patients had major complications. Sixty-four (56%) patients had minor complications during the first 12 months after surgery (i.e., both transient and permanent complications as defined per protocol). Twenty-six (23%) patients accounted for both major and minor complications. Forty-two (37%) patients did not have any complication at any time point. In 17 of the 33 patients (52%) patients the major complications had resolved at the 24 months follow-up, and in 30 of 64 (47%) patients the minor complications had resolved. Eighty-one (70%) patients did not have any remaining complications at the 24 months follow-up.

**Table 3:** Major complications after microvascular decompression (n = 115)

Complication	N (%) During the first 12 months	N (%) 24-months follow-up
Death	0	–
Infarction; cerebellar or brainstem	6 (5)	6 (5)
Haemorrhage; cerebellar or brainstem	1 (1)	1 (1)
Anaesthesia dolorosa	0^b^	0
Meningitis	0	0
Cerebrospinal fluid leak	6 (5)	0
Hydrocephalus	0	0
Permanent ataxia	8 (7)^c^	6 (5)
Permanent diplopia	0^d^	0
Corneal keratitis	2 (2)^e^	2 (2)
Permanent severe hypoesthesia	8 (7)^f^	7 (6)
Permanent facial weakness/nerve palsy	3 (3)^g^	3 (3)
Permanent hearing loss	1 (1)	1 (1)
Permanent hearing impairment	11 (10)	10 (9)

Values represent numbers of patients (%). Major complications were death, stroke, meningitis, cerebrospinal fluid leak, hydrocephalus, permanent ataxia, permanent diplopia, corneal keratitis, permanent severe hypoesthesia, anaesthesia dolorosa, permanent facial weakness/nerve palsy, permanent hearing loss and permanent hearing impairment

^a^Permanent was defined as persisting 12 months after microvascular decompression. The clinical course of all patients with major complications are described in detail in Table 5. Additional minor or major complications derived from stroke are not represented in this table nor in Table 4 with minor complications

^b^One stroke patient had anaesthesia dolorosa as a sequalae after an infarct

^c^All patients who suffered a stroke had permanent ataxia

^d^Three patients who suffered a stroke had permanent diplopia

^e^Two patients who suffered a stroke had corneal keratitis

^f^One patient who suffered a stroke had permanent and severe hypoesthesia

^g^One patient who suffered a stroke had permanent facial weakness

**Table 4:** Minor complications after microvascular decompression (n = 115)

Complication	N (%) Lasting less than 12 months	N (%) At 12-months follow-up	N (%) At 24-months follow-up
Dizziness	5 (4)	4 (4)^c^	3 (3)
Tinnitus	1 (1)	4 (4)^d^	2 (2)
Tiredness	1 (1)	2 (2)^e^	1 (1)
Wound infection	3 (3)	0	0
Headache	6 (5)	5 (4)	3 (3)
Scar tissue pain	8 (7)	9 (8)	6 (5)
Transient^a^ ataxia	7 (6)	NA	NA
Transient^a^ diplopia	5 (4)	NA	NA
Permanent^b^ mild hypoesthesia	NA	12 (10)^f^	7 (6)
Transient^a^ mild hypoesthesia	6 (5)	NA	NA
Transient^a^ severe hypoesthesia	0	NA	NA
Trigeminal motor weakness (masseter dysfunction)	2 (2)	2 (2)^g^	0
Transient^a^ facial weakness	0	NA	NA
Transient^a^ hearing loss	0	NA	NA
Transient^a^ hearing impairment	6 (5)	NA	NA
Altered sense of taste	0	6 (5)^h^	6 (5)

Values represent numbers of patients (%) unless otherwise indicated. Minor complications were dizziness, tinnitus, tiredness, wound infection, headache, scar tissue pain, transient ataxia, transient diplopia, permanent mild hypoestesia, transient mild hypoesthesia, transient severe hypoesthesia, trigeminal motor weakness, transient facial nerve palsy, transient hearing loss, transient hearing impairment and altered sense of taste

^a^Transient was defined as lasting less than 12 months

^b^Permanent was defined as persisting 12 months after microvascular decompression. NA: Not applicable as it is considered a major complication, see Table 3. The clinical course of all patients with major complications are described in detail in Table 5. Additional minor or major complications derived from stroke are not represented in this table nor in Table 3 with major complications

^c^Three patients who suffered a stroke had permanent dizziness which persisted at 24-months follow-up

^d^One patient who suffered a stroke had permanent tinnitus which had resolved at 24-months follow-up

^e^One patient who suffered a stroke had permanent tiredness which persisted at 24-months follow-up

^f^Two patients who suffered a stroke had permanent mild hypoesthesia which persisted at the 24-month follow-up

^g^Two stroke patients had permanent trigeminal motor weakness, which persisted at the 24-months follow-up

^h^One stroke patient had permanent altered sense of taste, which persisted at the 24-months follow-up

**Table 5:** The peri- and postoperative course of patients with major complications following microvascular decompression

Patient no.	Description of peri- and postoperative complications following microvascular decompression^a^
1^b^	The procedure was technically difficult as the compressing superior cerebellar artery was difficult to uncover because it emitted a branch that ran between the trigeminal motor branch and the trigeminal sensory branch. Therefore, the trigeminal nerve was decompressed with 3 pieces of Teflon. During surgery, there was brief arterial bleeding from a small arteriole from a branch of the compressing artery. The bleeding was stopped using bipolar coagulation. A postoperative MRI^c^ displayed *infarction in the right side of the pons and cerebellum* ipsilateral to the operated side. The resulting complications were *ataxia, diplopia, anaesthesia dolorosa* in all 3 branches of the trigeminal nerve, recurrent *keratitis,* and *reduced cornea sensibility,* which all persisted at the 12- and 24 months follow-up. The modified Rankin Scale score was 3. The surgical outcome at 24 months was “Failure” (BNI VA).
2^b^	The procedure itself was uncomplicated. Postoperatively, the patient had *diplopia, dizziness, headache* and *scar tissue pain*. A postoperative MRI displayed discrete *infarction in the right side of the pons* ipsilateral to the operated side. The resulting complications were *permanent ataxia, permanent diplopia* and *permanent dizziness* which persisted at the 24 months follow-up. The modified Rankin Scale score was 1. The surgical outcome at 24 months was “Failure” (BNI V A).
3^b^	The procedure was technically difficult due to limited exposure and venous bleeding from the petrosal vein which was stopped by bipolar coagulation. A postoperative MRI displayed *infarction in the right side of the pons* ipsilateral to the operated side. The resulting complications were *ataxia*, *diplopia, dizziness, post-craniotomy headache, extreme tiredness, tinnitus and trigeminal motor weakness* which all persisted at the 12- and 24 -months follow-up. The Modified Rankin Scale score was 1. The surgical outcome at 24 months was “Excellent” (BNI I).
4^b^	The procedure itself was uncomplicated. A postoperative MRI displayed *infarction in the left side of the pons* ipsilateral to the operated side. The resulting symptoms of *ataxia, facial weakness* and *hemiparesis* which persisted at the 12 months follow-up. At the 24 months follow-up, the only persisting complication was *discrete ataxia*. The Modified Rankin Scale score was 1. Initially the patient was completely pain free, but 2 months postoperatively there was a recurrence of pain with the same characteristics and location as preoperatively. The surgical outcome at 24 months was “Failure” as the patient had re-surgery (balloon compression) 12 months after MVD.
5 ^b^	The procedure was technically difficult as the compressing vein was situated in between the trigeminal motor branch and the trigeminal sensory branch of the trigeminal nerve and compressed the trigeminal nerve in three different places. Furthermore, the surgeon had to manipulate with the left superior cerebellar artery as it also encountered the trigeminal nerve and was adherent to the nerve by thickened arachnoid. A postoperative MRI displayed *infarction in the left side of the pons*, *cerebellum and the cerebellar peduncle* ipsilateral to the operated side*.* The resulting complications were *ataxia, and trigeminal motor weakness.* There was also *mild hypoesthesia* of the left side of the face and intraorally and *subjective cognitive deficits* such as lack of initiative and overview. All complications persisted at the 12- and 24 months follow-up. The Modified Rankin Scale score was 2. The surgical outcome at 24 months was “Excellent” (BNI I).
6^b^	The procedure was complicated as the superior cerebellar artery had several branches that was situated right by the root entry zone and were difficult to mobilize. Furthermore, a small vein that ran between the trigeminal motor branch and the trigeminal sensory branch was coagulated. A large vein was also translocated away from the trigeminal nerve and a piece of Teflon was interposed. Altogether two pieces of Teflon was used. A postoperative MRI displayed *small infarcts scattered in the right side of the cerebellum and the right side of the pons* ipsilateral to the operated side. The patient had *transient diplopia* as well as *ataxia* and *altered sense of taste* which persisted at the 12- and 24-months follow-up. The Modified Rankin Scale score was 1. The surgical outcome at 24 months was “Good” (BNI IIIA).
7 ^b^	The procedure itself was uncomplicated. A postoperative MRI displayed an *acute haemorrhage* at the right cerebellar peduncle ipsilaterally to the operated trigeminal nerve. The resulting complications were *ataxia, dizziness, mild hypoesthesia,* and *allodynia* of all trigeminal branches which persisted at the 12 months follow-up. At the 24 months follow-up the patient had *allodynia, mild hypoesthesia, ataxia, dizziness, corneal keratitis, and tinnitus.* The Modified Rankin Scale score was 1. The surgical outcome *was “Excellent”* (BNI I).
8	The procedure itself was uncomplicated. Immediately postoperatively, the patient had *rhinoliquorrhea. Initially, it was treated conservatively with bed rest, but due to continuous rhinoliquorrhea the patient eventually had a lumbar drain which was not sufficient either. Finally, at re-operation a small* cerebrospinal fluid fistula *was re-sutured. There were no complications at the 12- and 24 months follow-up. The surgical outcome* at 24 months *was “Excellent” (BNI I).*
9	The procedure itself was uncomplicated. Seven days postoperatively the patient had *CSF leakage from the scar*. At re-operation, a small cerebrospinal fluid fistula was re-sutured. The wound heeled with no further complications apart from *occasional headache*. At 24 months follow-up, there were no complications. The surgical outcome at 24 months was “Excellent” (BNI I).
10	The procedure itself was uncomplicated. Two days postoperatively the patient had *CSF leakage from the scar*. An extra suture was placed, and the leakage stopped. *There were no complications at 12- and 24 months follow-up.* *The surgical outcome* at 24 months *was “Excellent” (BNI I).*
11	The procedure itself was uncomplicated. Postoperatively, the patient had *CSF leakage from the wound* and *rhinoliquorrhea*. At re-operation a cerebrospinal fluid fistula at approximately 1 cm × 2 cm was closed with a net and titanium screws. Two months after re-operation the patient had *scar tissue pain* which persisted at the 12 months follow-up. Nineteen months after the MVD the patient had a re-operation where the net and screws were removed. It reduced but did not eliminate the *scar tissue pain* which persisted at the 24 months follow-up. The surgical outcome at 24 months was “Poor” (BNI IV).
12	The procedure itself was uncomplicated. Postoperatively, the patient had *CSF leakage from the wound* and *wound drainage that was suspected to be purulent.* The patient was treated with i.v. antibiotics and re-operated where a small cerebrospinal fluid fistula was re-sutured. The wound healed with no further complications. The resulting complications where a constant *headache* which persisted at 12- and 24 months follow-up. The surgical outcome at 24 months was “Failure” (BNI VA).
13	The procedure itself was uncomplicated. Postoperatively, the patient had *CSF leakage from the wound.* At re-operation a cerebrospinal fluid fistula was re-sutured. The patient had no further complications at 12- and 24 months follow-up. The surgical outcome at 24 months was “Excellent” (BNI I).
14	The procedure itself was uncomplicated. Postoperatively, the patient had *severe hypoesthesia* and *hearing impairment* ipsilateral to the operated side that persisted at the 12- and 24 months follow-up. The patient had a *herpes zoster keratitis* as a complication to the *loss of corneal sensitivity*. This eventually resulted in *loss of vision* on the right eye 18 months after the procedure. The surgical outcome at 24 months was “Failure” (BNI VB).
15	The procedure itself was uncomplicated. Postoperatively, the patient had *blurred vision* and an ophthalmologist confirmed the diagnosis of *keratoconjunctivitis sicca* as a surgical complication due to *reduced cornea sensibility*. Furthermore, the patient had *reduced tear production and severe hypoesthesia* in the left 1st and 2nd trigeminal branch which persisted at the 12-months and 24 -months follow-up. The surgical outcome at 24 months was “Excellent” (BNI I).
16	The procedure itself was uncomplicated. Postoperatively, the patient had *ataxia* that persisted at the 12- and 24 months follow-up. The surgical outcome at 24 months was “Excellent” (BNI I).
17	The procedure was technically difficult as there was a large sclerotic vertebral artery loop which compressed and dislocated the 7th and 8th cranial nerve and came into contact with the trigeminal nerve at the root entry zone. A postoperative MRI months after the procedure displayed gliosis in the left middle cerebellar peduncle. The resulting complications were *permanent ataxia, permanent left facial nerve paralysis* (resulting in lagophthalmos which was treated with implantation of platin in the left eyelid), *permanent hearing impairment*, *permanent dizziness* and *eating difficulties* which persisted at 12 months follow-up. All complications except eating difficulties persisted also at 24 months follow-up. The surgical outcome at 24 months was “Excellent” (BNI I).
18	The procedure itself was uncomplicated. The patient had *transient dizziness*, *hearing impairment, mild hypoesthesia of the right side of the face, tinnitus and altered sense of taste* that persisted at the 12- and 24 months follow-up. The surgical outcome at 24 months was “Excellent” (BNI I).
19	The procedure itself was uncomplicated. Postoperatively, the patient had *hypoesthesia*, *hearing impairment* on the left ear and *dizziness*. The patient refused to undergo MRI. A postoperative CT could not detect any infarction or haemorrhage. The resulting complications were *permanent ataxia, permanent severe hypoesthesia, permanent hearing impairment and permanent dizziness.* All complications persisted at the 24 months follow-up, apart from the hypoesthesia which now was mild. The surgical outcome at 24 months was “Good” (BNI II).
20	The procedure itself was uncomplicated. Postoperatively, the patient had *ataxia, mild hypoesthesia and hearing impairment* which persisted at the 12 months follow-up. At the 24 months follow-up the ataxia had resolved, and the remaining complications were *mild hypoesthesia and hearing impairment*. The surgical outcome at 24 months was “Excellent” (BNI I).
21	The procedure itself was uncomplicated. Postoperatively, the patient had *transient mild hypoesthesia* and *ataxia* that persisted at the 12 months follow-up. At 24 months follow-up the only complication was scar tissue pain. The surgical outcome at 24 months was “Excellent” (BNI I).
22	The procedure itself was uncomplicated. Postoperatively, the patient had *hearing impairment, ataxia* and *dizziness* and *scar tissue pain* which all persisted at the 12 months follow-up. At the 24 months follow-up all complications except hearing impairment persisted. The surgical outcome at 24 months was “Good” (BNI II).
23	The procedure itself was uncomplicated. Postoperatively, the patient had *severe hypoesthesia* that persisted at the 12- and 24 months follow-up. The surgical outcome at 24 months was “Excellent” (BNI I).
24	The procedure was technically difficult as there close to the end of the procedure was a venous bleeding of app. 3000 mL from the branches of the superior petrosal vein. A post-operative MRI could not detect any infarction or haemorrhage. The patient had *transient ataxia, transient diplopia, severe hypoesthesia, hearing impairment, dizziness, tiredness* and *altered sense of taste* that persisted at the 12 months follow-up. All complications except tiredness also persisted at the 24 months follow-up. The surgical outcome at 24 months was “Failure” (BNI VA).
25	The procedure itself was uncomplicated. Postoperatively, the patient had *severe hypoesthesia* and *altered sense of taste* that persisted at the 12- and 24 months follow-up. The surgical outcome at 24 months was “Excellent” (BNI I).
26	The procedure itself was uncomplicated. Postoperatively, the patient had *severe hypoesthesia* and *altered sense of taste* that persisted at the 12- and 24 months follow-up. The surgical outcome at 24 months was “Excellent” (BNI I).
27	The procedure itself was uncomplicated. Postoperatively, the patient had *severe hypoesthesia* and *altered sense of taste* that persisted at the 12- and 24 months follow-up. In addition, the patient had developed *corneal keratitis* at the 24 months follow-up. The surgical outcome at 24 months was “Good” (BNI II).
28	The procedure itself was uncomplicated. Postoperatively, the patient had a worsening of pre-existing *paraesthesia* (due to previous glycerol injection and balloon compression), *transient trigeminal motor weakness* and *permanent facial nerve palsy.* The facial nerve palsy persisted at 24 months follow-up. The surgical outcome at 24 months was “Failure” (BNI VB).
29	The procedure itself was uncomplicated. A postoperative MRI could not detect any infarction or haemorrhage. The patient had *ataxia, facial nerve palsy, hearing loss, dizziness, tinnitus,* and *trigeminal motor weakness* which persisted at 12 months follow-up. The patient had a platin weight inserted in the upper eye lid. *Ataxia, facial nerve palsy and hearing loss* persisted at the 24 months follow-up. The surgical outcome at 24 months was “Poor” (BNI IV).
30	The procedure itself was uncomplicated. Postoperatively, the patient had *transient diplopia* and *hearing impairment* that persisted at the 12- and 24 months follow-up. The surgical outcome was “Failure” as the patient had a balloon compression 4 months and again 7 months after the procedure.
31	The procedure was technically difficult due to limited exposure. Postoperatively, the patient had *transient tinnitus* and *hearing impairment* that persisted at the 12- and 24 months follow-up. The surgical outcome at 24 months was “Excellent” (BNI I).
32	The procedure itself was uncomplicated. Postoperatively, the patient had *mild hypoesthesia* and *hearing impairment* ipsilateral to the operated side. The mild hypoesthesia persisted at 12 months follow-up but was gone at 24 months follow-up. The *hearing impairment* persisted at 24 months follow-up. The surgical outcome at 24 months was “Excellent” (BNI I).
33	The procedure itself was uncomplicated. Postoperatively, the patient had *transient dizziness* and *hearing impairment* that persisted at the 12- and 24 months follow-up. The surgical outcome was “Excellent” (BNI I).

*BNI* Barrow Neurological Institute

*MVD* microvascular decompression

^a^ if nothing is noted on the technical procedure, there was no apparent injury to the cranial nerve VII/VIII complex

^b^Stroke after microvascular decompression

^c^A postoperative MRI was only performed if there was clinical sign of complications

### Major complications

**Fig. 3:**
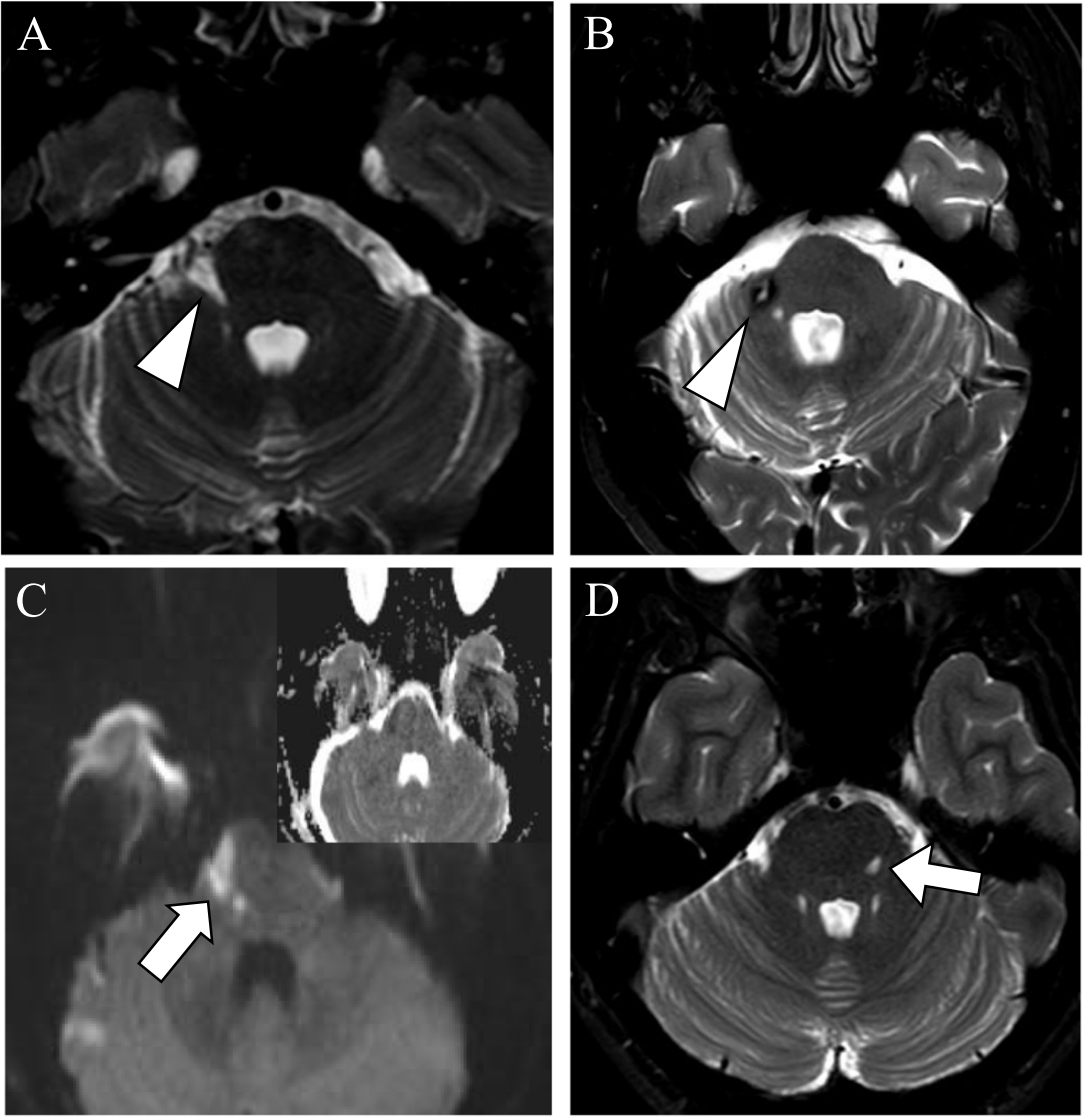
Postoperative MRIs of patients with stroke after microvascular decompression. Postoperative MRI of patients after MVD. (a) axial T2 DRIVE weighted sequence shows chronic infarction (arrowhead) in the right cerebellar peduncle of patient no 1 (Supplementary material B). (b) axial T2 DRIVE weighted sequence shows sequelae after hemorrhage (arrowhead) at the right cerebellar peduncle of patient no. 7. c) axial diffusion-weighted sequence shows subacute infarction (arrow) in the right side of the pons of patient no. 3. (d) axial T2 DRIVE weighted sequence shows chronic infarction (arrow) in the left side of the pons in patient no. 4

Seven (6%) patients had a stroke (Fig. 3). Of these, one (1%) patient had a postoperative haemorrhagic stroke at the level of pons and six (5%) patients had ischemic strokes. Of the latter, three (50%) patients had a pontine infarction, one patient had an infarct in the pons and cerebellum, one patient had infarction involving the pons, the cerebellar peduncle and the cerebellum, and one patient had multiple infarcts scattered in both the cerebellum and the pons. In four of the seven cases the procedure was complicated by difficult anatomy (Additional file 2: Supplementary material B).

All seven patients with stroke had persisting symptoms at the 24 months follow-up (Additional file 2: Supplementary material B). Based on the Modified Rankin Scale, 5 (71%) patients with stroke could continue their normal life as they scored a “1” (no significant disability despite symptoms; able to carry out all usual duties and activities), one patient scored “2” (slight disability; unable to carry out all previous activities but able to look after own affairs without assistance) and 1 patient scored “3” (moderate disability; requiring some help but able to walk without assistance).

### Association between major complications, outcome, age and surgical characteristics

Out of the 33 (29%) patients with major complications, 23 (70%) patients had a clinically significant outcome. Forty (35%) patients had only minor complications and of those, 36 patients (90%) had a clinically significant outcome. Of the 42 (37%) patients without any complications at any time point after the MVD, 40 patients (95%) had a clinically significant outcome. Patients with major complications, had a significantly higher prevalence of a poor or failed surgical outcome compared to patients without major complications (10 (30%) vs. 6 (7%), *p* = 0.004). Of the 33 patients with major complications 18 (55%) patients had a NVC with morphological changes. There was no difference in the prevalence of major complications in the two groups (classical TN with major complications (18/52) vs. idiopathic TN with major complications (15/30), *p* = 0.378). In seven (21%) of the 33 patients the procedure was technically complicated (Additional file 2: Supplementary material B).

There was no significant difference between the prevalence of major complications in the patients above 70 years (13 (39%)) compared to the patients aged 70 years and under (20 (38%)), *p* = 0.874.

The prevalence of patients that had perioperative coagulation of the petrosal vein was similar in those with major complications and those without (i.e. none or minor complications) (17 (52%) vs. 42 (51%) *p* = 0.977).

### Patient satisfaction and patient recommendation

All patients returned the self-complete questionnaire. One hundred and eight (94%) patients reported that they would recommend MVD to other patients. Ninety-one (79%) patients replied that they were satisfied, and 17 (15%) patients replied that they were dissatisfied with their current situation. Of the 33 (29%) patients with major complications, 27 (82%) would still recommend MVD to others and 17 (52%) were satisfied with their current situation. The high level of satisfaction may mirror the high degree of disability caused by the intense pain prior to surgery [26]. This association between high patient satisfaction and a failed outcome has also been shown by Barker et al. [14]

## Discussion

With state-of-the-art methodology we show that most patients with TN have a clinically significant outcome after MVD. The rates of complications were higher than previously reported. Still, the degree of disability was low in most patients with stroke, and most patients would recommend MVD to others, irrespective of complications, indicating that most of the complications were acceptable for the patients as surgery led to significant pain relief.

Our findings support the notion that MVD should be first choice surgery in classical TN but add important nuances to this picture as the chance of an excellent outcome is dependent on the sex and the degree of NVC. We show, for the first time, that major complications are related to a poor outcome.

### Outcome after microvascular decompression

A prospective study including 164 patients undergoing first-time surgical treatment reported pain freedom after MVD in 83% at 1 year and 61% at 5 years follow-up [27] Two smaller prospective studies reported pain freedom in 68% of 24 included patients at 12 months follow-up [28] and pain freedom in 88% of 36 of the included patients at the 24 months follow-up [18]. Thus, our findings are generally in line with previous studies.

A prospective study of 1336 patients and a retrospective study including 372 patients with long-term follow-up show that the majority of recurrences take place in the first 2 years after MVD [13, 14]. This may indicate that looking at outcomes 2 years after surgery is clinically important, and that most of the patients in this study will have a high chance of continuing being pain-free, in the future.

### Excellent outcome is associated to neurovascular contact and male sex

We show that an excellent outcome is associated to both NVC with morphological changes and the male sex. Other studies also found a strong association between a NVC with morphological changes and a good surgical outcome [29, 30]. Barker et al. showed that female sex was a significant predictor of eventual recurrence [14]. Yet other studies did not find sex to be a significant predictor of a favourable outcome [18, 31] This study was not powered to investigate differences in outcome between women and men, but our data indicate the women have poorer outcome than men. However, this need to be confirmed in other larger studies before firm conclusions can be made. Outcome of MVD in women must in future multi-center studies be compared to percutaneous procedures. Diffusion tensor imaging studies show that pathological abnormalities at the root entry zone of the trigeminal nerve in patients with TN resolves after MVD [32]. This was interpreted as a sign of remyelination at the site of the NVC [32]. This could explain why a NVC with morphological changes is highly associated to a better outcome [13, 30].

MVD appears to be effective in idiopathic TN with NVC without morphological changes, though not to the same degree as in classical TN. Invasive procedures have a high placebo response, [33] but we consider it unlikely that the placebo effect alone can account for the documented high efficacy of surgery. NVC without morphological changes can be part of a polyfactorial disease aetiology in patients with idiopathic TN [34].

We found that men had better outcome after MVD [13]. This could suggest that TN aetiology in men is mainly monofactorial and mostly dependent on NVC, whereas the aetiology in women may be more complex and polyfactorial. We did not find that the presence of concomitant continuous pain was related to outcome which is supported by another study [35].

### Complication rates after microvascular decompression

This study uses independent assessors of outcome and complications after MVD in patients with TN, and for comparison of results on complications, we have selected studies mostly adhering to the essential criteria developed Zakrzewska et al. [36] when reporting surgical studies in TN, [13, 14, 18, 28, 31, 37] and disregarded others with a clear risk of bias or major methodological shortcomings and hence carrying a high risk of overestimating efficacy and underestimating complication rate.

Ten % had permanent hearing impairment and 1% had hearing loss. Similar rates of hearing loss were seen in previous studies [13, 14, 37].. Yet, another study found that using perioperative brainstem auditory evoked potential reduced hearing complications, at least for untrained surgeons [38].. We report that 6% of patients had MRI-verified stroke as a result of MVD. This is similar to another study using independent assessors of complications who reported a complication rate of stroke of 4.3% [31]. Other studies report no strokes at all [18, 28, 37] or much lower rates (0.7% -1.54%) [14, 39]. Fortunately, the disability caused by stroke was mild in the majority of patients in this study. The mild disability could explain why stroke was not reported in some studies. Our findings on hypoesthesia are somewhat comparable to previous studies reporting hypoesthesia in 5–10% of patients [13, 14, 18, 37].

A prospective multi-center study evaluating complications and effect of 166 patients with TN who had undergone MVD found that 16.3% had complications at day 7 postoperatively and that only in 5.2% the complications were permanent. However, it remains unclear how this assessment was done, and to which extend the patient was involved as the authors only refer to “investigators” that recorded complications. Furthermore the complications were grouped in rather broad categories rendering the results less transparent [40].

Coagulation of the petrosal vein was not significantly associated to major complications contrary to what one might expect. Studies on this topic are ambiguous, as some studies find an increased prevalence of complications when sacrificing the petrosal vein [41] and others do not [23, 42]. In some cases, petrosal vein sacrifice can be necessary to permit a wider access to the root entry zone of the trigeminal nerve, and venous sacrifice is typically limited to cases where it is deemed necessary for successful trigeminal nerve decompression.

A study indicated that one must retain a high volume of MVDs per surgeon to maintain operating skills as morbidity rates were significantly lower at high-volume hospitals with high-volume surgeons [43]. The specific neurosurgical unit involved in this study is one of the two neurosurgical departments in Denmark where MVD is performed. The three neurosurgeons who did MVDs in this unit all have a long surgical experience including a very high degree of specialization into skull base surgery and vascular neurosurgery. Finally, we confirm previous studies indicating that MVD is safe in the elderly [44].

We are primed to report more complications, especially transient and minor complications with a protocol with predefined possible complications [45] In addition, we can not rule out that complication rate is related to the surgical technique.

### Patient satisfaction

Despite that MVD carries a risk of potentially serious complications, our study shows that almost all patients would recommend the procedure to other patients — even those patients who suffered major complications. This may reflect the high chance of a clinically significant outcome as well as the high degree of disability caused by the intense pain [26]. Based on our findings and combined with clinical experience, we argue that while there is a documented risk of surgical complications after MVD, this risk should be weighed against the high chance of a clinically relevant surgical outcome combined with the weight of the excruciating and intense pain and debilitating medical side effects rendering the patient severely affected.

### Strengths and limitations

This study is a strictly designed and systematically conducted prospective cohort study with well-characterized consecutive patients, preoperative 3.0 Tesla MRI evaluated blinded to the symptomatic side and preoperative evaluation of diagnosis and medical treatment by a neurologist. It is a major strength that evaluation of outcome and complications was made by a neurologist and not the operating neurosurgeon. The risk of a biased evaluation of efficacy and outcome was most likely considerably reduced by having neurologists evaluating the results in combination with patient-directed questionnaires.

With predefined complications we are primed to report more complications, especially minor and transient complications [45]. A longer term prospective follow-up is warranted to follow natural history and late recurrence. One could argue that the composite score of the BNI scale is not the most appropriate outcome in TN. However, at the outset of the study the BNI scale was considered suitable and easy to use in the clinic. For future studies, we would recommend to use patient-related outcomes measures, e.g., the Global Impression of Change scale or the Pen Facial Pain Scale Revised [46, 47]..

## Conclusions

MVD is an effective treatment for patients with classical and idiopathic (only patients with a neurovascular contact) TN. Surgical complications are relatively frequent warranting thorough patient selection and information preoperatively. MVD should be considered in patients with TN where medical treatment is ineffective or causing significant side effects. Future prospective multi-centered studies with independent assessors of outcome and complications are needed to further elucidate the effect and complications of MVD.

## Supplementary Information

**Additional file 1.** Supplementary material A 1: Self-complete questionnaire for surgical patients

**Additional file 2.** Supplementary material B. The peri- and postoperative course of patients with major complications following microvascular decompression.

## Data Availability

Data were collected at the DHC and can be made available from the corresponding author upon reasonable request and after approval from the Danish Data Protection Agency.

## Abbreviations

BNI: Barrow Neurological Institute
DHC: Danish Headache Center
ICHD: International Classification of Headache Disorders
NVC: neurovascular contact
NRS: numeric rating scale
MVD: Microvascular decompression
