# Supplementary material for: Microvascular decompression in trigeminal neuralgia - a prospective study of 115 patients
Source: J Headache Pain. 2022 Nov 19;23(1):145. doi: 10.1186/s10194-022-01520-x (PMC9675260; doi:10.1186/s10194-022-01520-x)
Supplement: Supplementary file 1 — Additional file 1. Supplementary material A 1: Self-complete questionnaire for surgical patients [file 10194_2022_1520_MOESM1_ESM.pdf]

Self-complete questionnaire for surgical patients

This questionnaire encompasses 31 questions concerning your last operation for trigeminal neuralgia and will be used by your doctor at the Danish Headache Center to see if you have benefited from the operation. Please bring the completed questionnaire with you on your next visit to the Danish Headache Center.

Thank you in advance for taking your time to complete the questionnaire.

Patient name:…………………….. Date of completion of questionnaire:…………
Name and date of birth:…………………. Date of operation:……………

Please circle the most appropriate answer.

This section is concerning your pain and whether you have experiences pain since your last operation.

1.	Have you had TN pain since your last operation?	No □ (go to Q12) Yes □
2a.	The pain you feel now, is that the same as before the surgery?	No □ Yes □ Do not know □
2b.	If the pain does not feel the same way as before the surgery, how does the pain feel different?	□ Still paroxysmal stabbing pain, but less intensive<br>□ Still paroxysmal stabbing pain, but more intensive<br>□ Constant/boring<br>□ Other, please describe:……………………………….
3.	When did you experience pain again after your surgery?	□ Immediately<br>□ 1-6 months after the surgery<br>□ 7-12 months after the surgery<br>□ 1-2 years after the surgery
4.	Is the pain present all the time?	□ No<br>□ Yes (go to Q7)
5.	If the pain is not there all the time, but comes and goes, how long does a pain attack lasts?	□ Seconds □ Minutes □ Hours □ Days
6.	How long does it usually take between the bouts of pain?	□ Minutes □ Hours □ Days □ Weeks
7.	What is the pain intensity?	0 1 2 3 4 5 6 7 8 9 10<br>No pain Worst imaginable pain
8.	Where is the pain localized?	□ in the same area as before the surgery<br>□ in another area than before the surgery
9.	What can trigger the pain?	□ Touch □ Talking<br>□ Brushing teeth □ Eating<br>□ Cold wind □ Chewing<br>□ Other, please describe:………………..
10.	Have you taken TN medication for your pain?	□ No (go to Q12) □ Daily use □ Sometimes
11.	What TN medication do you take for your TN pain?	Name of the medicine:……………………………<br>Dosage pr. day in mg:…………………….

This section is about whether you have experienced any complications after your last surgery for trigeminal neuralgia

12a	If you had any hypoesthesia before the surgery, has the surgery then changed it?	□ I have never experienced hypoesthesia<br>□ Yes, and it is worse than before<br>□ Yes, but its better now than before<br>□ Yes, but it is unchanged
12b	Have you after the surgery experienced less hypoesthesia in an area where you have experienced hypoesthesia before the surgery?	□ No (go to Q17)<br>□ Yes
13	If you answered yes in Q12b how long time after the operation did you experience hypoesthesia?	□ Immediately<br>□ 1-2 months after the surgery<br>□ 3-5 months after the surgery<br>□ 6 months or more after the surgery
14	How severe is the hypoesthesia?<br>(compared to for instance anaesthesia at the dentist)	□ No □ Mild □ Severe
15	How painful is it to touch the area with hypoesthesia?	□ No pain □ Mild pain □ Severe pain
16	Have you experienced a burning pain or a different sensation in the face ipsilateral to the side of operation?	□ Not at all □ Mild □ Severe
17	Have you experienced impaired hearing after the operation?	□ Not at all □ Mild □ Severe
18	If yes, has this impaired hearing changed in some way?	□ It lasted less than 6 months – but it is now back to normal<br>□ It lasted more than 6 months – but it is now back to normal

	☐ It is unchanged<br>☐ The hearing impairment is still there but it has improved.<br>☐ The hearing impairment is worse
19	Do you feel unsecure when walking after your latest operation? ☐ No ☐ Yes
20	Do you feel dizzier now than before you latest surgery? ☐ No ☐ Yes
21	Do you have more headache than usual after your latest surgery? ☐ No ☐ Yes
22	Have you after the operation had eating difficulties which you have not experienced before? ☐ No ☐ Yes
23	Have you had vision problems after the operation which you have not experienced before? ☐ No ☐ Yes
24	Have you experienced any complications after the operation which is not mentioned above?<br>If yes, please describe:………………………………………………………………
25	Please describe how the complications affect your quality of life?<br>Numbness: Not at all Slightly Moderately Pronounced<br>Burning sensation/changed sensation Not at all Slightly Moderately Pronounced<br>Dizziness Not at all Slightly Moderately Pronounced<br>Headache Not at all Slightly Moderately Pronounced<br>Eating difficulties Not at all Slightly Moderately Pronounced<br>Vision problems Not at all Slightly Moderately Pronounced<br>Other complications, please describe:……. Not at all Slightly Moderately Pronounced<br>…………………………………………….

The last section is about how your general view on the surgery and your patient satisfaction.

26.	When you look back, do you think that the time of surgery was correct? ☐ The surgery was done at the correct time<br>☐ The surgery should have been done earlier<br>☐ The surgery should have been done later<br>☐ The surgery should never have been performed.
27.	
28.	Do you experience that the TN pain restrain you in your activities with respect to your leisure time, work and/or education?<br>Yes No Not relevant<br>a. Work ☐ ☐ ☐<br>b. Education ☐ ☐ ☐<br>c. Leisure time ☐ ☐ ☐
29.	Have you during the last month felt down or depressed? ☐ No ☐ Yes
30.	Have you during the last month experienced reduced desire or interest in doing things you would normally want to do or feel interest in? ☐ No ☐ Yes
31.	Would you recommend this surgery to others? ☐ No ☐ Yes

THANK YOU - for completing the questionnaire.

If you have any further comments, feel free to write them on the back of this questionnaire.
