# Supplementary material for: Microvascular decompression in trigeminal neuralgia - a prospective study of 115 patients
Source: J Headache Pain. 2022 Nov 19;23(1):145. doi: 10.1186/s10194-022-01520-x (PMC9675260; doi:10.1186/s10194-022-01520-x)
Supplement: Supplementary file 2 — Additional file 2. Supplementary material B. The peri- and postoperative course of patients with major complications following microvascular decompression. [file 10194_2022_1520_MOESM2_ESM.docx]

| **Supplementary material B. The peri- and postoperative course of patients with major complications following microvascular decompression.** | |
| --- | --- |
| **Patient no.** | **Description of peri- and postoperative complications following microvascular decompression^a^** |
| **1^b^** | The procedure was technically difficult as the compressing superior cerebellar artery was difficult to uncover because it emitted a branch that ran between the trigeminal motor branch and the trigeminal sensory branch. Therefore, the trigeminal nerve was decompressed with 3 pieces of Teflon. During surgery, there was brief arterial bleeding from a small arteriole from a branch of the compressing artery. The bleeding was stopped using bipolar coagulation.  A postoperative MRI^c^ displayed *infarction in the right side of the pons and cerebellum* ipsilateral to the operated side. The resulting complications were *ataxia, diplopia, anaesthesia dolorosa* in all 3 branches of the trigeminal nerve, recurrent *keratitis,* and *reduced cornea sensibility,* which all persisted at the 12- and 24 months follow-up. The modified Rankin Scale score was 3. The surgical outcome at 24 months was “Failure” (BNI VA). |
| **2^b^** | The procedure itself was uncomplicated. Postoperatively, the patient had *diplopia, dizziness, headache* and *scar tissue pain*. A postoperative MRI displayed discrete *infarction in the right side of the pons* ipsilateral to the operated side. The resulting complications were *permanent* *ataxia, permanent diplopia* and *permanent dizziness* which persisted at the 24 months follow-up. The modified Rankin Scale score was 1. The surgical outcome at 24 months was “Failure” (BNI V A). |
| **3^b^** | The procedure was technically difficult due to limited exposure and venous bleeding from the petrosal vein which was stopped by bipolar coagulation.  A postoperative MRI displayed *infarction in the right side of the pons* ipsilateral to the operated side. The resulting complications were *ataxia*, *diplopia,* *dizziness, post-craniotomy headache, extreme tiredness, tinnitus and trigeminal motor weakness* which all persisted at the 12- and 24 -months follow-up. The Modified Rankin Scale score was 1. The surgical outcome at 24 months was “Excellent” (BNI I). |
| **4^b^** | The procedure itself was uncomplicated.  A postoperative MRI displayed *infarction in the left side of the pons* ipsilateral to the operated side. The resulting symptoms of *ataxia, facial weakness* and *hemiparesis* which persisted at the 12 months follow-up. At the 24 months follow-up, the only persisting complication was *discrete ataxia*. The Modified Rankin Scale score was 1. Initially the patient was completely pain free, but two months postoperatively there was a recurrence of pain with the same characteristics and location as preoperatively.  The surgical outcome at 24 months was “Failure” as the patient had re-surgery (balloon compression) 12 months after MVD. |
| **5 ^b^** | The procedure was technically difficult as the compressing vein was situated in between the trigeminal motor branch and the trigeminal sensory branch of the trigeminal nerve and compressed the trigeminal nerve in three different places. Furthermore, the surgeon had to manipulate with the left superior cerebellar artery as it also encountered the trigeminal nerve and was adherent to the nerve by thickened arachnoid.  A postoperative MRI displayed *infarction in the left side of the pons*, *cerebellum and the cerebellar peduncle* ipsilateral to the operated side*.* The resulting complications were *ataxia,* *and trigeminal motor weakness.* There was also *mild hypoesthesia* of the left side of the face and intraorally and *subjective* *cognitive deficits* such as lack of initiative and overview. All complications persisted at the 12- and 24 months follow-up. The Modified Rankin Scale score was 2. The surgical outcome at 24 months was “Excellent” (BNI I). |
| **6^b^** | The procedure was complicated as the superior cerebellar artery had several branches that was situated right by the root entry zone and were difficult to mobilize. Furthermore, a small vein that ran between the trigeminal motor branch and the trigeminal sensory branch was coagulated. A large vein was also translocated away from the trigeminal nerve and a piece of Teflon was interposed. Altogether two pieces of Teflon was used.  A postoperative MRI displayed *small infarcts scattered in the right side of the cerebellum and the right side of the pons* ipsilateral to the operated side. The patient had *transient diplopia* as well as *ataxia* and *altered sense of taste* which persisted at the 12- and 24-months follow-up. The Modified Rankin Scale score was 1. The surgical outcome at 24 months was “Good” (BNI IIIA). |
| **7 ^b^** | The procedure itself was uncomplicated.  A postoperative MRI displayed an *acute haemorrhage* at the right cerebellar peduncle ipsilaterally to the operated trigeminal nerve. The resulting complications were *ataxia, dizziness, mild hypoesthesia,* and *allodynia* of all trigeminal branches which persisted at the 12 months follow-up. At the 24 months follow-up the patient had *allodynia, mild hypoesthesia, ataxia, dizziness, corneal keratitis, and tinnitus.* The Modified Rankin Scale score was 1. The surgical outcome was “Excellent” (BNI I). |
| **8** | The procedure itself was uncomplicated. Immediately postoperatively, the patient had rhinoliquorrhea. Initially, it was treated conservatively with bed rest, but due to continuous rhinoliquorrhea the patient eventually had a lumbar drain which was not sufficient either. Finally, at re-operation a small cerebrospinal fluid fistula was re-sutured. There were no complications at the 12- and 24 months follow-up. The surgical outcome at 24 months was “Excellent” (BNI I). |
| **9** | The procedure itself was uncomplicated. Seven days postoperatively the patient had *CSF leakage from the scar*. At re-operation, a small cerebrospinal fluid fistula was re-sutured. The wound heeled with no further complications apart from *occasional headache*. At 24 months follow-up, there were no complications. The surgical outcome at 24 months was “Excellent” (BNI I). |
| **10** | The procedure itself was uncomplicated. Two days postoperatively the patient had *CSF leakage from the scar*. An extra suture was placed, and the leakage stopped. There were no complications at 12- and 24 months follow-up.  The surgical outcome at 24 months was “Excellent” (BNI I). |
| **11** | The procedure itself was uncomplicated. Postoperatively, the patient had *CSF leakage from the wound* and *rhinoliquorrhea*. At re-operation a cerebrospinal fluid fistula at approximately 1 cm x 2 cm was closed with a net and titanium screws. Two months after re-operation the patient had *scar tissue pain* which persisted at the 12 months follow-up. Nineteen months after the MVD the patient had a re-operation where the net and screws were removed. It reduced but did not eliminate the *scar tissue pain* which persisted at the 24 months follow-up.  The surgical outcome at 24 months was “Poor” (BNI IV). |
| **12** | The procedure itself was uncomplicated. Postoperatively, the patient had *CSF leakage from the wound* and *wound drainage that was suspected to be purulent.* The patient was treated with i.v. antibiotics and re-operated where a small cerebrospinal fluid fistula was re-sutured. The wound healed with no further complications. The resulting complications where a constant *headache* which persisted at 12- and 24 months follow-up.  The surgical outcome at 24 months was “Failure” (BNI VA). |
| **13** | The procedure itself was uncomplicated. Postoperatively, the patient had *CSF leakage from the wound.* At re-operation a cerebrospinal fluid fistula was re-sutured. The patient had no further complications at 12- and 24 months follow-up.  The surgical outcome at 24 months was “Excellent” (BNI I). |
| **14** | The procedure itself was uncomplicated. Postoperatively, the patient had *severe hypoesthesia* and *hearing impairment* ipsilateral to the operated side that persisted at the 12- and 24 months follow-up. The patient had a *herpes zoster keratitis* as a complication to the *loss of corneal sensitivity*. This eventually resulted in *loss of vision* on the right eye 18 months after the procedure.  The surgical outcome at 24 months was “Failure” (BNI VB). |
| **15** | The procedure itself was uncomplicated. Postoperatively, the patient had *blurred vision* and an ophthalmologist confirmed the diagnosis of *keratoconjunctivitis sicca* as a surgical complication due to *reduced cornea sensibility*. Furthermore, the patient had *reduced tear production and severe hypoesthesia* in the left 1^st^ and 2^nd^ trigeminal branch which persisted at the 12-months and 24 -months follow-up.  The surgical outcome at 24 months was “Excellent” (BNI I). |
| **16** | The procedure itself was uncomplicated. Postoperatively, the patient had *ataxia* that persisted at the 12- and 24 months follow-up.  The surgical outcome at 24 months was “Excellent” (BNI I). |
| **17** | The procedure was technically difficult as there was a large sclerotic vertebral artery loop which compressed and dislocated the 7^th^ and 8^th^ cranial nerve and came into contact with the trigeminal nerve at the root entry zone.  A postoperative MRI months after the procedure displayed gliosis in the left middle cerebellar peduncle. The resulting complications were *permanent ataxia, permanent left facial nerve paralysis* (resulting in lagophthalmos which was treated with implantation of platin in the left eyelid), *permanent* *hearing impairment*, *permanent* *dizziness* and *eating difficulties* which persisted at 12 months follow-up. All complications except eating difficulties persisted also at 24 months follow-up.  The surgical outcome at 24 months was “Excellent” (BNI I). |
| **18** | The procedure itself was uncomplicated. The patient had *transient dizziness*, *hearing impairment, mild hypoesthesia of the right side of the face, tinnitus and altered sense of taste* that persisted at the 12- and 24 months follow-up.  The surgical outcome at 24 months was “Excellent” (BNI I). |
| **19** | The procedure itself was uncomplicated. Postoperatively, the patient had *hypoesthesia*, *hearing impairment* on the left ear and *dizziness*. The patient refused to undergo MRI. A postoperative CT could not detect any infarction or haemorrhage. The resulting complications were *permanent ataxia, permanent severe hypoesthesia, permanent hearing impairment and permanent dizziness.* All complications persisted at the 24 months follow-up, apart from the hypoesthesia which now was mild.  The surgical outcome at 24 months was “Good” (BNI II). |
| **20** | The procedure itself was uncomplicated. Postoperatively, the patient had *ataxia, mild hypoesthesia and hearing impairment* which persisted at the 12 months follow-up. At the 24 months follow-up the ataxia had resolved, and the remaining complications were *mild hypoesthesia and hearing impairment*.  The surgical outcome at 24 months was “Excellent” (BNI I). |
| **21** | The procedure itself was uncomplicated. Postoperatively, the patient had *transient mild hypoesthesia* and *ataxia* that persisted at the 12 months follow-up. At 24 months follow-up the only complication was scar tissue pain.  The surgical outcome at 24 months was “Excellent” (BNI I). |
| **22** | The procedure itself was uncomplicated. Postoperatively, the patient had *hearing impairment, ataxia* and *dizziness* and *scar tissue pain* which all persisted at the 12 months follow-up. At the 24 months follow-up all complications except hearing impairment persisted.  The surgical outcome at 24 months was “Good” (BNI II). |
| **23** | The procedure itself was uncomplicated. Postoperatively, the patient had *severe hypoesthesia* that persisted at the 12- and 24 months follow-up.  The surgical outcome at 24 months was “Excellent” (BNI I). |
| **24** | The procedure was technically difficult as there close to the end of the procedure was a venous bleeding of app. 3000 mL from the branches of the superior petrosal vein.  A post-operative MRI could not detect any infarction or haemorrhage.  The patient had *transient ataxia, transient diplopia,* *severe hypoesthesia, hearing impairment, dizziness, tiredness* and *altered sense of taste* that persisted at the 12 months follow-up. All complications except tiredness also persisted at the 24 months follow-up.  The surgical outcome at 24 months was “Failure” (BNI VA). |
| **25** | The procedure itself was uncomplicated. Postoperatively, the patient had *severe hypoesthesia* and *altered sense of taste* that persisted at the 12- and 24 months follow-up.  The surgical outcome at 24 months was “Excellent” (BNI I). |
| **26** | The procedure itself was uncomplicated. Postoperatively, the patient had *severe hypoesthesia* and *altered sense of taste* that persisted at the 12- and 24 months follow-up. The surgical outcome at 24 months was “Excellent” (BNI I). |
| **27** | The procedure itself was uncomplicated. Postoperatively, the patient had *severe hypoesthesia* and *altered sense of taste* that persisted at the 12- and 24 months follow-up. In addition, the patient had developed *corneal keratitis* at the 24 months follow-up.  The surgical outcome at 24 months was “Good” (BNI II). |
| **28** | The procedure itself was uncomplicated. Postoperatively, the patient had a worsening of pre-existing *paraesthesia* (due to previous glycerol injection and balloon compression), *transient trigeminal motor weakness* and *permanent facial nerve palsy.* The facial nerve palsy persisted at 24 months follow-up.  The surgical outcome at 24 months was “Failure” (BNI VB). |
| **29** | The procedure itself was uncomplicated. A postoperative MRI could not detect any infarction or haemorrhage. The patient had *ataxia, facial nerve palsy, hearing loss, dizziness, tinnitus,* and *trigeminal motor weakness* which persisted at 12 months follow-up. The patient had a platin weight inserted in the upper eye lid. *Ataxia, facial nerve palsy and hearing loss* persisted at the 24 months follow-up.  The surgical outcome at 24 months was “Poor” (BNI IV). |
| **30** | The procedure itself was uncomplicated. Postoperatively, the patient had *transient diplopia* and *hearing impairment* that persisted at the 12- and 24 months follow-up.  The surgical outcome was “Failure” as the patient had a balloon compression 4 months and again 7 months after the procedure. |
| **31** | The procedure was technically difficult due to limited exposure.  Postoperatively, the patient had *transient tinnitus* and *hearing impairment* that persisted at the 12- and 24 months follow-up.  The surgical outcome at 24 months was “Excellent” (BNI I). |
| **32** | The procedure itself was uncomplicated. Postoperatively, the patient had *mild hypoesthesia* and *hearing impairment* ipsilateral to the operated side. The mild hypoesthesia persisted at 12 months follow-up but was gone at 24 months follow-up. The *hearing impairment* persisted at 24 months follow-up.  The surgical outcome at 24 months was “Excellent” (BNI I). |
| **33** | The procedure itself was uncomplicated. Postoperatively, the patient had *transient dizziness* and *hearing impairment* that persisted at the 12- and 24 months follow-up.  The surgical outcome was “Excellent” (BNI I). |
| BNI: Barrow Neurological Institute  MVD: microvascular decompression  **^a^** if nothing is noted on the technical procedure, there was no apparent injury to the cranial nerve VII/VIII complex.  ^b^Stroke after microvascular decompression.  ^c^A postoperative MRI was only performed if there was clinical sign of complications. | |
